# Supplementary material for: Perceptual error based on Bayesian cue combination drives implicit motor adaptation
Source: eLife. 2024 Jul 4;13:RP94608. doi: 10.7554/eLife.94608 (PMC11223768; doi:10.7554/eLife.94608)
Supplement: Supplementary file 1. — (a) Model fitting and simulation parameters with the PEA model. (b) Model comparisons. [file elife-94608-supp1.docx]

**Supplementary File 1a.** Model fitting and simulation parameters with the PEA model.

|  | Data set | Parameters | | | | | | Goodness-of-fit | |
| --- | --- | --- | --- | --- | --- | --- | --- | --- | --- |
|  |  | $\sigma_{u}$  (deg) | $\sigma_{p}$  (deg) | *a* | *b* | *A* | *B* | *R^2^* | *RMSE*  (deg) |
| Adaptation extent fitting  (Figure 1B) | Kim 2018, Exp1 | -- | $\sigma_{p}$*/a* = 3.406; *b/a* = 0.138 | | | -- | -- | 0.773 | 1.898 |
|  | Kim 2018, Exp2 | -- | $\sigma_{p}$*/a* = 4.758; *b/a* = 0.168 | | | -- | -- | <0 | 2.163 |
|  | Morehead, 2017 | -- | $\sigma_{p}$*/a* = 1.639; *b/a* = 0.044 | | | -- | -- | <0 | 2.937 |
| Trial-by-trial adaptation fitting | Exp 2,  Figure 3 | 5.048 | 11.119 | *1.853 | *0.309 | 0.970 | 0.208 | 0.975 | 1.222 |
|  |  | 5.468 | 12.128 | 1.663 | 0.331 | 0.971 | 0.194 | 0.975 | 1.217 |
|  | Exp 2,  Figure 3-figure supplement 4 | -- | -- | -- | -- | 0.991 | 0.364 | 0.989 | 0.815 |
|  | Tsay 2019, Figure 4A | 1.896 | 7.959 | *1.853 | *0.309 | 0.978 | 0.525 | 0.991 | 0.716 |
| Single-trial learning fitting | Tsay 2021, Figure 3-figure supplement 3 | $\sigma_{int}$ *=* 7.364 | | 1.179 | 0.384 | -- | 0.057 | 0.974 | 0.020 |
| Proprioceptive recalibration simulation | Exp 3,  Figure 4B | 5.048 | 11.119 | 1.853 | 0.309 | 0.970 | 0.208 | -- | -- |
| Adaptation affected by visual uncertainty simulation | Exp 4,  Figure 5 | 5.048 | 11.119 | 1.853 | 0.309 | -- | 0.208 | -- | -- |

* Asterisks represent fixed parameters in specific data fitting. The fixed values equal to the slope and intercept estimated from Experiment 1.

**Supplementary File 1b.** Model comparisons.

| Data set |  | PEA | PReMo | Causal Inference |
| --- | --- | --- | --- | --- |
| Block-design learning fitting  Exp 2, Figure 3 & S3 | AIC | 2255 | 3543 | 3283 |
|  | *R^2^* | 0.975 | 0.749 | 0.711 |
|  | *RMSE* (deg) | 1.222 | 3.896 | 4.151 |
| Single-trial learning fitting  Tsay 2021, Figure 3-figure supplement 3 | AIC | -36.90 | -15.98 | -11.28 |
|  | *R^2^* | 0.974 | 0.584 | 0.305 |
|  | *RMSE* (deg) | 0.020 | 0.103 | 0.080 |
